# B-mode Ultrasound Characteristics of Thyroid Nodules With High-Benign Probability and Nodules With Risk of Malignancy

**DOI:** 10.7759/cureus.39281

**Published:** 2023-05-20

**Authors:** Salahaden R Sultan

**Affiliations:** 1 Radiologic Sciences, Faculty of Applied Medical Sciences, King Abdulaziz University, Jeddah, SAU

**Keywords:** neck imaging, tirads, thyroid nodule, b-mode, ultrasound

## Abstract

Introduction: Thyroid nodules are commonly found on clinical examination or diagnostic imaging of the neck. Malignant thyroid nodules are increasing worldwide, making thyroid cancer one of the most common endocrine malignancies worldwide. The aim of this study was to determine B-mode ultrasound characteristics of benign thyroid nodules and nodules with risk of malignancy.

Material and methods: This retrospective study was conducted on subjects (n=99) who underwent thyroid ultrasound. Data were retrieved from the Thyroid Digital Image Database of Universidad Nacional de Colombia, a published open-access dataset, in which B-mode ultrasound images were interpreted by expert radiologists providing a complete diagnostic description of thyroid lesions using the Thyroid Imaging Reporting and Data System.

Results: Sponge-like appearance (Pearson Chi-Square 4.6, p=0.02), cystic (Pearson Chi-Square 27.3, p<0.001), isoechoic (Pearson Chi-Square 26, p<0.001), and well-defined (Pearson Chi-Square 13.7, p<0.001) thyroid nodules were more likely to be observed in benign nodules (risk of malignancy <5%). On the other hand, predominately solid (Pearson Chi-Square 5.9, p=0.01), microcalcifications (Pearson Chi-Square 50.7, p<0.001), hypoechoic (Pearson Chi-Square 27.7, p<0.001), irregular shape (Pearson Chi-Square 6.6, p=0.01), and ill-defined (Pearson Chi-Square 8.8, p=0.003) thyroid nodules were more likely to be observed in nodules with risk of malignancy (>5%).

Conclusion: Ultrasound characteristics could be used to determine thyroid nodules with risk of malignancy and avoid over-diagnosing nodules with benign features. Further research evaluating the use of multiparametric ultrasound to distinguish between benign thyroid nodules and thyroid nodules with risk of malignancy is required.

## Introduction

Thyroid nodules, discrete lesions within the thyroid gland, are common incidental findings on clinical examination. It has been reported that the prevalence of thyroid nodules may be found in 50% of the adult population [1], with less than 15% of all thyroid nodules being clinically relevant with potential risk of thyroid cancer [2,3]. Although thyroid nodules are commonly asymptomatic and are benign and clinically insignificant [4], malignant nodules continue to grow with annual increasing trends worldwide up to 15%, making thyroid cancer one of the most common endocrine malignancies worldwide and being seventh and 15th most common cancer in women and men, respectively [5,6]. Therefore, differentiating between benign and malignant thyroid nodules using a non-invasive method is important.

Incidental thyroid nodules can be also detected with the use of diagnostic imaging of the neck for purposes unrelated to the thyroid. Ultrasound is the first-line imaging examination for the identification of thyroid nodules [7] and has improved the malignancy risk assessment of thyroid nodules cancer through sonographic findings, including assessment of the nodule echogenicity, internal composition, calcification and border regularity [5,8]. In addition, patient characteristics including age and gender have been reported to be associated with increased risk factor of thyroid cancer in which thyroid cancer predominately affects women, but may have higher mortality in men and worse prognosis in older age [2]. 

Ultrasound-guided fine-needle aspiration cytology (FNA) and core-needle biopsy (CNB) are considered gold standards for pre-operative diagnosis and are performed in patients with suspected malignancy [9,10]. However, these procedures are not required for benign thyroid nodules and should be performed on patients with thyroid nodules of increased risk of malignancy. For this, the aim of this study was to identify characteristics of thyroid nodules on ultrasound images as an attempt to determine thyroid nodules with risk of malignancy that would benefit from FNA and/or CNB, and avoid over-diagnosis of benign nodules.

## Materials and methods

Data acquisition

**Figure 1:**
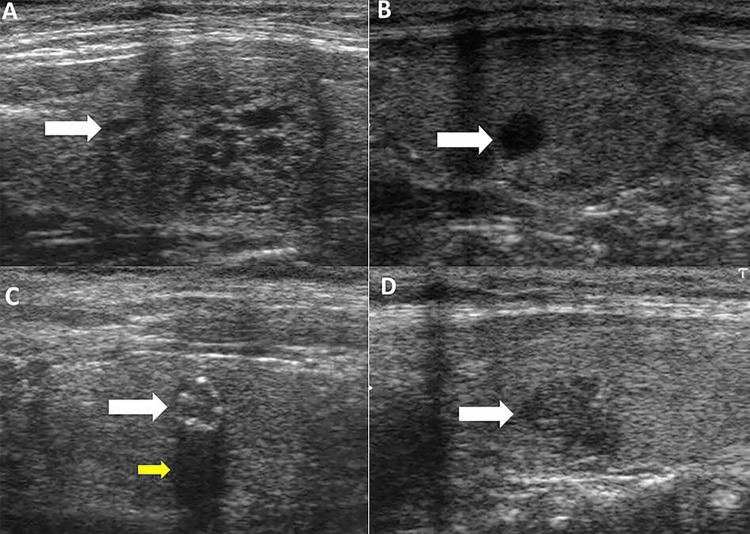
B-mode ultrasound characteristics of thyroid nodules. Isoechoic well-defined sponge-like appearance nodule (white arrow, A); Anechoic well-defined cystic nodule (white arrow, B); Hypoechoic solid well-defined nodule with micro-calcification (white arrow), causing acoustic shadowing (yellow arrow, C); Hypoechoic solid nodule with irregular margin (white arrow, D).

This observational retrospective study was conducted on subjects who underwent thyroid ultrasound examination. Data were retrieved from the Thyroid Digital Image Database of Universidad Nacional de Colombia, a published open-access dataset, in which B-mode ultrasound images were interpreted by expert radiologists providing a complete annotation and diagnostic description of thyroid lesions using the Thyroid Imaging Reporting and Data System (TIRADS) [11,12]. No ethical approval was required. In this study, only data from adult subjects with information on age, gender, and a complete diagnostic description of thyroid nodules features, including nodule appearance and composition (sponge-like appearance, solid/predominately solid mass, cystic mass and microcalcification), echogenicity compared to the normal surrounding tissues of thyroid parenchyma (hypoechoic, isoechoic, hyperechoic, and mixechogenicity), shape (round, oval and irregular), and nodule margin (well-defined and ill-defined) were extracted (Figure 1). Thyroid nodules were divided into two categories: high-benign probability with very low risk of malignancy (<5%) for nodules diagnosed as TIRADS I, II and III, and with risk of malignancy (≥5%) for nodules diagnosed as TIRADS IV and V [12].

Statistical analysis

A Chi-square test was used for comparison of categorical variables, that is, the association between each thyroid nodule's features in B-mode ultrasound and the diagnosis of the nodules. Data analysis was performed using Statistical Package for Social Sciences (SPSS) version 21.0 (IBM Corp., Armonk, NY, USA) and PRISM 7 (GraphPad Software, La Jolla, CA, USA). Statistical significance was set at p< 0.05. 

## Results

Ninety-nine patients compromising 33 probably benign thyroid nodules and 66 with risk of malignancy were included in the present study. There was a significant relationship between gender and presence of thyroid nodules, with females significantly higher than males (p<0.001). No significant difference in age between patients with high-benign probability and those with risk of malignant thyroid nodules (patients with probably benign nodules, 54.2±17.2; patients with high-risk malignant nodules, 54.4±15.9, mean age±standard deviation).

B-mode ultrasound characteristics of thyroid nodules

**Figure 2:**
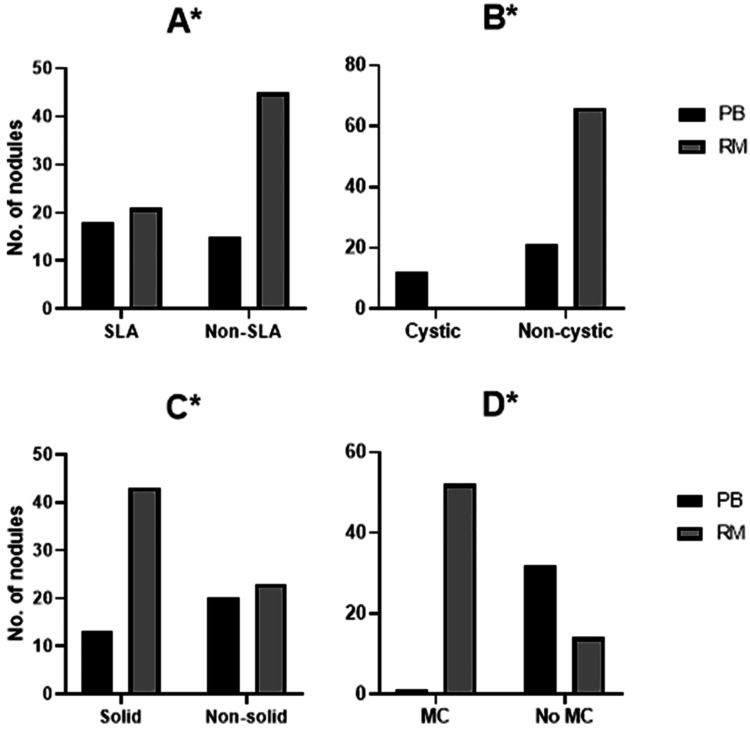
Probably benign (PB) and risk of malignancy (RM) thyroid nodule composition. Nodules with sponge-like appearance (SLA) vs non-SLA (A), cystic vs cystic (B), solid vs non-solid (C), and microcalcification (MC) vs no MC (D). *p<0.05 using chi-square test (number of nodules (n)=99, probably benign n=33 and risk of malignancy n=66).

Thyroid nodular composition showed significant differences between probably benign nodules and nodules with risk of malignancy. Sponge-like appearance of thyroid nodules (Pearson Chi-Square 4.6, p=0.02, Figure 2A) and nodules with cystic structures (Pearson Chi-Square 27.3, p<0.001, Figure 2B) were more likely to be observed in probably benign nodules than in nodules with risk of malignancy, with 54.5% to 31.8% and 36.4% to 0%, respectively. On the other hand, solid or predominately solid nodules (Pearson Chi-Square 5.9, p=0.01, Figure 2C) and nodules with microcalcifications (Pearson Chi-Square 50.7, p<0.001, Figure 2D) were more likely to be observed in nodules with risk of malignancy than in probably benign nodules, with 65.2% to 39.4% and 78.8% to 3%, respectively.

**Figure 3:**
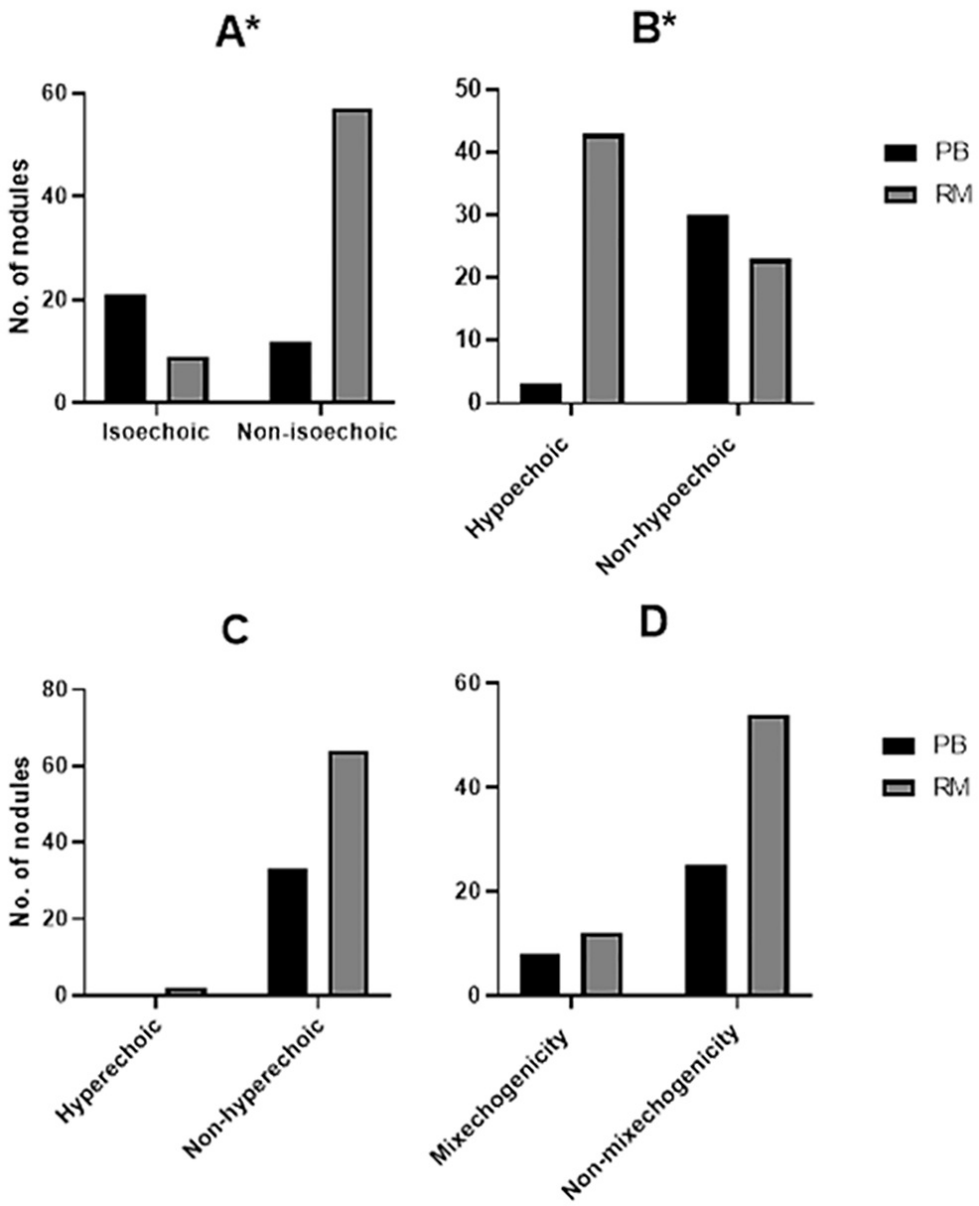
Echogenicity of probably benign (PB) and risk of malignancy (RM) thyroid nodules compared to normal surrounding thyroid tissue. Isoechoic vs non-isoechoic (A), hypoechoic vs non-hypoechoic (B), hyperechoic vs non-hyperechoic (C), and mixechogenicity vs non-mixechogenicity (D). * p<0.05 using chi-square test (number of nodules (n)=99, probably benign n=33 and risk of malignancy n=66).

When comparing thyroid nodule echogenicity to normal surrounding tissue, isoechoic nodules were significantly associated with being probably benign and nodules with risk of malignancy were hypoechoic (isoechoic nodule: Pearson Chi-Square 26, p<0.001, Figure 3A; hypoechoic nodule: Pearson Chi-Square 27.7, p<0.001, Figure 3B). Isoechoic nodules were more likely to be observed in probably benign nodules than in nodules with risk of malignancy, with 63.6% to 13.6%; whereas hypoechoic nodules were more likely to be observed in nodules with risk of malignancy than in probably benign nodules, with 65.2% to 9.1%. No association between hyperechoic (p=0.31, Figure 3C) or mixechogenicity (p=0.47, Figure 3D) of the thyroid nodule when compared to normal tissue and the nature of the nodule. 

**Figure 4:**
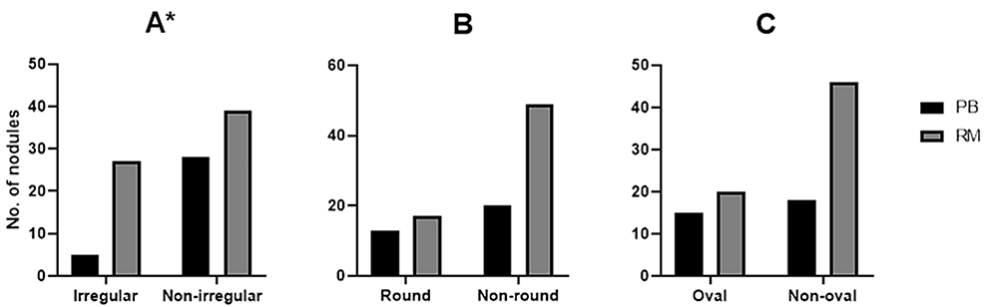
Thyroid nodule shape of probably benign (PB) and risk of malignancy (RM). Nodules with irregular vs non-irregular (A), round vs non-round (B), and oval vs non-oval (C) shape. * p<0.05 using chi-square test (number of nodules (n)=99, probably benign n=33 and risk of malignancy n=66).

Irregular shape of thyroid nodules was significantly associated with risk of malignancy nodules than in probably benign nodules, with 40.9% to 15.2% (Pearson Chi-Square 6.6, p=0.01, Figure 4A), but no associations between round or oval shape nodule and the nature of the nodule (round shape, p=0.16, Figure 4B; oval shape, p=0.13, Figure 4C).

**Figure 5:**
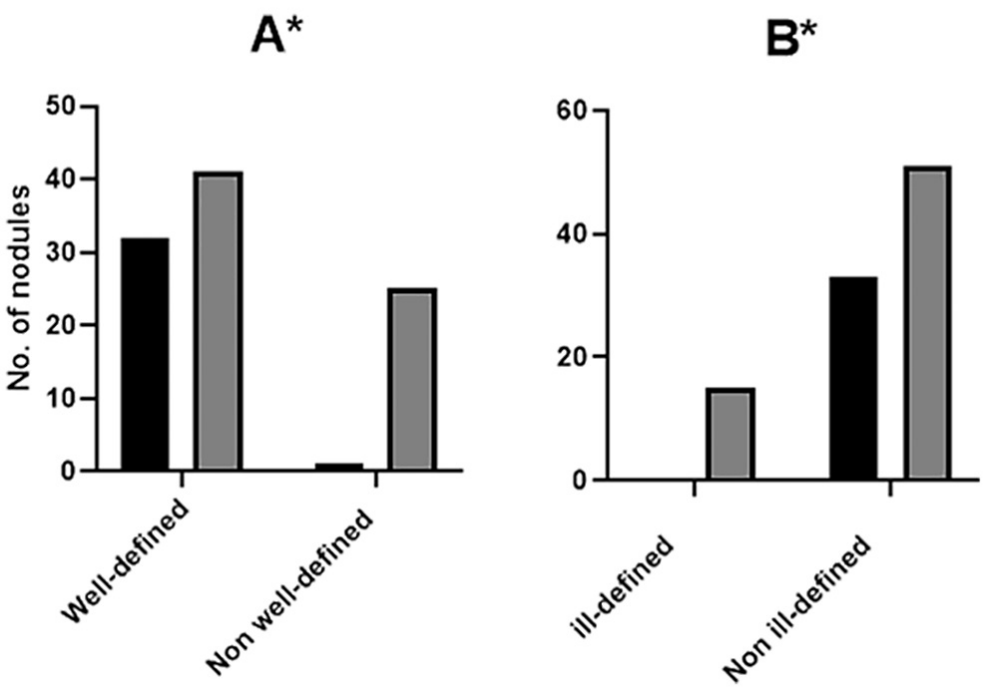
Margin of probably benign (PB) and risk of malignancy (RM) thyroid nodules. Well-defined vs non-well-defined (A) and ill-defined vs non-ill-defined (B). * p<0.05 using chi-square test (number of nodules (n)=99, probably benign n=33 and risk of malignancy n=66).

Well-defined and ill-defined nodules margins were significantly associated with being probably benign and with risk of malignancy, respectively (well-defined: Pearson Chi-Square 13.7, p<0.001, Figure 5A; ill-defined: Pearson Chi-Square 8.8, p=0.003, Figure 5B). Well-defined nodules were more likely to be observed in probably benign nodules than in nodules with risk of malignancy, with 97% to 62.1%; whereas ill-defined nodules were more likely to be observed in nodules with risk of malignancy than in probably benign nodules, with 22.7% to 0%.

## Discussion

The present study investigated thyroid nodule characteristics on B-mode ultrasound images as an attempted to determine thyroid nodules with risk of malignancy that would benefit from FNA and/or CNB for treatment decision. Predominately solid, hypoechoic, presence of microcalcifications, with ill-defined and irregular shape were features of high-risk cancerous thyroid nodules. Conversely, benign features of thyroid nodules include nodules of predominately cystic, isoechoic and with well-defined margins. These ultrasound characteristics could be used to determine thyroid nodules with risk of malignancy to be considered for FNA and/or CNB pre-surgical treatment and avoid over-diagnosing nodules with benign features.

An initial diagnosis of thyroid is complex and requires accurate and precise methods for optimal treatment. However, due to the fact that thyroid nodules are commonly asymptomatic and detected incidentally through clinical examination or cross-sectional imaging, the diagnosis of thyroid nodules has been oversimplified, affecting treatment decision, and may lead to overtreatment [2,4]. B-mode ultrasound imaging is the primary tool for cancer risk stratification of thyroid nodules. An ultrasound diagnostic report should include description of the thyroid parenchyma, nodule location and size, number of nodules if multiple are detected, and features of cervical lymph nodes in addition to the thyroid nodule sonographic features [13]. Similar to our findings, it has been reported that thyroid nodules associated with malignancy on B-mode ultrasound images include solid composition, hypoechoic, irregular margins, and the presence of microcalcifications, in addition, nodules with calcified edge/rim are likely to be infiltrative cancer [14,15]. On the other hand, cystic and spongiform appearance of nodules are likely benign with a risk to be cancerous of <2% [14,15]. Furthermore, it has been reported that 5-10% of hyperechoic solid non-calcified thyroid are cancer [16]. However, in the present study, no association was found between the thyroid nodules of hyperechoic or mixechogenicity and the nature of the nodule. Moreover, patient characteristics including age and gender, and laboratory blood tests such as serum thyroid stimulating hormone and thyroid peroxidase are factors associated with risk of malignancy [2]. These suggest that the patient’s clinical characteristics and laboratory blood tests need to be considered besides sonographic features of thyroid nodules for optimal diagnosis. 

Heterogeneity and various echogenicity patterns of thyroid nodules on ultrasound images may make the diagnosis of thyroid cancer through visual assessment a challenging task, especially for inexperienced reporting sonographers or physicians. In addition, variability between observers may be present affecting the impression of the outcome diagnostic report [17,18]. It has been proposed that quantitative grey-scale analysis could improve the diagnosis rate and may be a useful tool to discriminate benign nodules from those with risk of malignancy, thus helping to reduce unnecessary FNA/CNB procedures [7]. Future studies investigating the use of quantitative grey-scale analysis alongside visual assessment and their correlation with FNA/CNB as gold standards are required.

Advanced ultrasound imaging methods could add more useful parameters to differentiate benign from malignant thyroid nodules [19-22]. The echogenicity of thyroid nodules is an important factor that reflects their cellular structure and composition. Subjective visual analysis is commonly used for nodule assessment, but it is limited by observer variability and experience. Quantitative gray-scale analysis can provide an objective measurement of nodule echogenicity and internal structure. It has been shown that the ultrasound gray-scale ratio, calculated from thyroid nodules and surrounding normal tissues, is a useful tool for differentiating between benign and malignant nodules in which malignant nodule is significantly lower than that of benign nodules [19,23]. However, caution should be considered when performing gray-scale analysis on thyroid nodules, as the echo intensity of nodules may vary depending on their size [24,25]. Ultrasound elastography is a noninvasive technique that can assess the stiffness of tissues and can be used in conjunction with gray-scale ultrasound, and may have potential for distinguishing benign and malignant thyroid nodules [26]. A recent meta-analysis of 11 randomized controlled trials evaluated the diagnostic value of ultrasound elastography in identifying benign and malignant 1,616 thyroid nodules from 1,333 patients using biopsy pathological diagnosis as the gold standard reported that ultrasound elastography has high sensitivity and specificity in diagnosing benign and malignant thyroid nodules [22]. It has also been reported that ultrasound elastography can effectively identify thyroid nodules that are likely to be noncancerous, reducing fine needle aspiration biopsy, and that thyroid nodules with low-risk features but high stiffness should be considered for fine needle aspiration biopsy [27]. Furthermore, the use of contrast-enhanced ultrasound (CEUS) may play an important role in identifying thyroid cancers by evaluating tumor microcirculation [28]. Hypoenhancement, heterogenicity and neovascularization are common indicators of malignancy [29]. A meta-analysis performed by Zhang et al. (2020) to evaluate the overall diagnostic value of CEUS for the characterization of thyroid nodules reported that CEUS might be a promising method for identifying malignancies from benign thyroid nodules [20]. Although these findings highlight the potential of ultrasound elastography and contrast-enhanced ultrasound as a valuable diagnostic tool in the clinical setting for thyroid nodules, it's worth noting that some benign nodules exhibit similar enhancement patterns and shearwave elastography values to malignant nodules [28], therefore, multiparametric ultrasound may offer new possibilities for preoperative distinction between benign and malignant thyroid nodules [30]. 

Limitations of this retrospective study include: evaluation of thyroid nodule ultrasound characteristics in real time was not possible due to the nature of the design of the study. The data set has been collected from a multicenter in which a nonuniform protocol of thyroid assessment may be followed; this may lead to variability of diagnostic reporting and nodules chosen for investigation. Further prospective studies investigating the correlation of ultrasound features and FNA/CNB are required.

## Conclusions

B-mode ultrasound characteristics of thyroid nodules with risk of malignancy include solid or predominately solid, hypoechoic, presence of microcalcifications, with ill-defined and/or irregular shape. Conversely, benign features of thyroid nodules include nodules of predominately cystic, isoechoic and with well-defined margins. These ultrasound characteristics could be used to determine thyroid nodules with risk of malignancy and avoid over-diagnosing nodules with benign features. Further research evaluating the use of multiparametric ultrasound to distinguish between benign and malignant thyroid nodules is required.
